# Stream acidification and reduced aquatic prey availability are associated with dietary shifts in an obligate riparian Neotropical migratory songbird

**DOI:** 10.7717/peerj.5141

**Published:** 2018-07-06

**Authors:** Brian K. Trevelline, Tim Nuttle, Brady A. Porter, Nathan L. Brouwer, Brandon D. Hoenig, Zachary D. Steffensmeier, Steven C. Latta

**Affiliations:** 1 Department of Biological Sciences, Duquesne University, Pittsburgh, PA, United States of America; 2 Civil and Environmental Consultants, Inc., Pittsburgh, PA, United States of America; 3 Department of Conservation and Field Research, National Aviary, Pittsburgh, PA, United States of America

**Keywords:** DNA metabarcoding, EPT, Louisiana Waterthrush, Macroinvertebrates, Next-generation sequencing, Resource subsidies

## Abstract

Streams and their surrounding riparian habitats are linked by reciprocal exchanges of insect prey essential to both aquatic and terrestrial consumers. Aquatic insects comprise a large proportion of total prey in riparian habitats and are opportunistically exploited by terrestrial insectivores; however, several species of songbirds are known to preferentially target aquatic prey via specialized foraging strategies. For these songbirds, reduced availability of aquatic insects via stream acidification may result in compensatory changes in provisioning during the nesting period, thereby influencing both adult and nestling diet composition. In this study, we used DNA metabarcoding to test the hypothesis that an obligate riparian Neotropical migratory songbird, the Louisiana Waterthrush (*Parkesia motacilla*), expands its diet to compensate for the loss of preferred aquatic prey taxa (primarily pollution-sensitive Ephemeroptera, Plecoptera, and Trichoptera) as a result of stream acidification. Our results revealed that both adult and nestling waterthrush exhibited an increase in dietary richness and niche breadth resulting from the consumption of terrestrial prey taxa in acidified riparian habitats. In contrast, compensatory dietary shifts were not observed in syntopic Neotropical migrant species known to primarily provision terrestrial prey taxa. In addition to providing support for our hypothesis that waterthrush compensate for stream acidification and aquatic prey limitations by expanding their diet, our findings highlight the vulnerability of Louisiana Waterthrush to anthropogenic disturbances that compromise stream quality or reduce the availability of pollution-sensitive aquatic insects.

## Introduction

Streams and their surrounding riparian habitats are linked by reciprocal exchanges of insect prey essential to both aquatic and terrestrial consumers (Baxter, Fausch & Carl Saunders, 2005; Polis, Anderson & Holt, 1997). Invertebrates with aquatic larval stages comprise a large proportion of total prey in riparian habitats (Nakano & Murakami, 2001) and are opportunistically exploited by birds during breeding (e.g., Gray, 1993; Rosenberg, Ohmart & Anderson, 1982; Trevelline et al., 2018), often resulting in a more diverse and densely populated avian community compared to adjacent non-riparian habitats (reviewed in Baxter, Fausch & Carl Saunders, 2005). In addition to avian species that opportunistically prey upon aquatic invertebrates, several passerines are known to preferentially target aquatic prey via specialized foraging strategies (e.g., Mattsson et al., 2009; Tyler & Tyler, 1996; Wilson & Kingery, 2011). Therefore, these stream-dependent birds may be highly vulnerable to land-use changes that disrupt the availability of aquatic invertebrates.

The availability of aquatic arthropods as prey for stream-dependent songbirds is largely determined by both chemical and geomorphic factors, some of which may be strongly influenced by anthropogenic activities (Rosenberg & Resh, 1993). For example, anthropogenic disturbances to riparian habitats such as abandoned mine discharge (Tomkiewicz & Dunson, 1977), acid precipitation (Graveland, 1998), hydraulic fracture (Wood, Frantz & Becker, 2016), thermal pollution (Benke, 1993), and urbanization (Roy et al., 2003) have been shown to alter the composition of riparian insect communities by reducing availability of pollution-sensitive larval and emergent aquatic taxa (particularly those in the orders Ephemeroptera, Plecoptera, and Trichoptera; hereafter EPT).

Riparian zones support several species of songbirds that are thought to specialize on pollution-sensitive EPT taxa (Mattsson et al., 2009; Ormerod & Tyler, 1991; Wilson & Kingery, 2011), and thus riparian habitats with reduced availability of these prey items support fewer breeding stream-dependent species compared to unimpacted drainages (Buckton et al., 1998; Feck & Hall, 2004; Mulvihill, Newell & Latta, 2008; Ormerod et al., 1986). Nevertheless, poor-quality riparian territories often remain occupied, typically by inexperienced breeders (Mulvihill, Newell & Latta, 2008). However, stream-dependent songbirds occupying acidified territories with reduced access to EPT may be at a disadvantage as evidenced by delayed clutch initiation (Mulvihill, Newell & Latta, 2008), smaller clutches (Ormerod et al., 1991), thinner egg shells (Ormerod et al., 1988), reduced nestling growth rate (Ormerod et al., 1991), lower nestling serum calcium levels (Ormerod et al., 1991), increased rates of nestling predation (O’Halloran et al., 1990), reduced nestling survival (Vickery, 1992), fewer nesting attempts (Mulvihill, Latta & Newell, 2009), and lower reproductive success (Price & Bock, 1983). All of these factors are thought to influence the annual breeding productivity of stream-dependent songbirds (e.g., Mattsson & Cooper, 2007), and migrants in general (reviewed in Martin, 1987). Therefore, reduced availability of EPT prey due to stream acidification may threaten the long-term conservation of birds that breed in riparian habitats.

For stream-dependent songbirds occupying anthropogenically degraded riparian habitats, the observed negative impacts to reproduction and nestling survival are typically associated with changes in foraging behavior. For example, Louisiana Waterthrush (*Parkesia motacilla*) nesting in acidified riparian habitats with reduced EPT availability expand their breeding territories and forage along unimpacted peripheral tributaries more frequently (Mulvihill, Newell & Latta, 2008). Similar behavioral responses have been observed in stream-dependent dippers (genus *Cinclus*), where individuals breeding in degraded habitats expand their foraging areas (Wilson & Kingery, 2011), spend more time away from the nest (O’Halloran et al., 1990), and feed nestlings less frequently (Vickery, 1992). These behavioral changes are thought to be part of a compensatory response to reduced availability of EPT taxa (Mulvihill, Newell & Latta, 2008; O’Halloran et al., 1990), which have been shown to be important prey during the period of nestling care (Mattsson et al., 2009; Trevelline et al., 2016; Trevelline et al., 2018). For non-riparian species of migratory songbirds, such changes in foraging behavior are typically accompanied by a concomitant shift in diet (e.g., Cooper et al., 1990; Sample, Cooper & Whitmore, 1993); however, it is unclear how stream-dependent songbirds alter their diets in response to stream acidification and reduced EPT availability.

In this study, we used DNA barcoding and next-generation sequencing (hereafter DNA metabarcoding) to investigate dietary shifts in a stream-dependent Neotropical migratory songbird, the Louisiana Waterthrush. We hypothesized that Louisiana Waterthrush occupying territories with reduced pH and EPT availability compensate by instead targeting terrestrial prey, thereby expanding their dietary niche.

## Materials and Methods

### Study species and sample collection

The Louisiana Waterthrush is a riparian-obligate passerine (family Parulidae) that nests directly in the banks of headwater mountain streams and primarily forages for aquatic insects (both larval and adult) in riffles and along stream edges (∼90% of foraging maneuvers directed at water, but occasionally glean insects from foliage; Mattsson et al., 2009). Louisiana Waterthrush is a species of conservation concern due to its dependence on high quality riparian areas and aquatic invertebrates that are sensitive to changes in water quality (Mattsson et al., 2009; Prosser & Brooks, 1998).

We measured stream pH using a handheld multi-parameter instrument (YSI Inc., Yellow Springs, OH, USA) at consistent locations within known waterthrush breeding territories (consistently occupied each breeding season) along three headwater streams at our long-term study sites near Powdermill Nature Reserve (Laurel Run, Loyalhanna Creek, and Powdermill Run; Westmoreland County, PA, USA) from April to June 2014 and 2015. These measurements were used to calculate the mean pH of each waterthrush territory over a two-year period, which were then used in subsequent statistical models to investigate dietary shifts in response to stream acidification.

To assess differences in prey availability between waterthrush territories, emergent aquatic insects taxa were continuously collected (at pH monitoring locations) throughout the entire 2015 breeding season (April–June) using sticky traps (Olson Products Inc., Medina, OH, USA; Collier & Smith, 1995). Because Louisiana Waterthrush are known to target both larval and emergent life-stages of aquatic arthropods (Mattsson et al., 2009), our characterization of EPT availability during the period of nestling care also included larval-stage benthic macroinvertebrates (collected using a D-frame dip net; Barbour et al., 1999). All EPT taxa collected via sticky traps and benthic sampling (300 individuals ± 20%; Barbour et al., 1999) were taxonomically identified to family using the diagnostic morphological characteristics provided by Merritt & Cummins (2008). The availability of EPT taxa (the total number of EPT individuals in sticky traps and benthic samples collected within 1 week of waterthush egg hatching divided by the total number of individuals) for each waterthrush territory was used in subsequent statistical models to investigate dietary shifts in response to EPT availability. Like Mulvihill, Newell & Latta (2008), we excluded the acid-tolerant families Leuctridae and Nemouridae (order Plecoptera) from our estimates of EPT availability to specifically assess the impact of stream acidification on the diet of Louisiana Waterthrush.

Nestling fecal samples were collected by placing nestlings (4–8 days old) into a clean paper bag (for up to 3 min) or by encouraging voidance directly over an open 20 mL vial of 100% ethanol. When possible, nestling fecal samples were collected on a second occasion 1–2 days later. Adults associated with each nest were captured using targeted mist-netting and briefly (3–5 min) placed into a clean paper bag lined with a clean 1-quart plastic bag (left open) to facilitate collection of fecal material. Adult fecal material was transferred from plastic bags into a 20 mL vial using a sterile serological pipette and 100% ethanol. All fecal samples were stored at −20 °C for approximately three months prior to DNA extraction.

All animal protocols were approved by the Institutional Animal Care and Use Committee of the National Aviary, Pittsburgh Zoo, and PPG Aquarium (approval reference number NA16-001). Permissions to complete fieldwork were provided by the Pennsylvania Game Commission, the Pennsylvania Department of Conservation and Natural Resources, and the USGS Bird Banding Lab. Access to study sites and logistical support were provided by the PA Bureau of Forestry, PA Bureau of State Parks, Powdermill Nature Reserve, and private landowners.

### Molecular analysis and bioinformatics

DNA was extracted using a protocol optimized for metabarcoding from avian fecal samples (Trevelline et al., 2016) and amplified using polymerase chain reaction (PCR) and general arthropod primers designed to target a 157 bp region of the mitochondrial cytochrome *c* oxidase I (COI) barcoding gene (Zeale et al., 2011). PCR amplification was performed in duplicate for each fecal sample (Trevelline et al., 2016; but see justification for triplicate PCR in Vo & Jedlicka, 2014) and pooled for an additional indexing reaction using the Illumina Nextera XT (v2) Indexing Kit following the manufacturer’s instructions (Trevelline et al., 2016). Once indexed, amplicons were pooled at equimolar concentrations and submitted to the Genomics Facility of the Biotechnology Resource Center at Cornell University (Ithaca, NY, USA) for analysis (250 bp paired-end) using the Illumina MiSeq next-generation sequencing platform. Illumina sequencing was performed using a loading concentration of 8 pM and 15% PhiX to improve the overall quality of the sequencing run given the low-complexity of our amplicon library.

Raw Illumina sequence reads were trimmed and quality filtered (Phred ≥ 30) using CLC Genomics Workbench 7.0.3 (Qiagen) and Galaxy 15.10 (Blankenberg et al., 2010; Giardine et al., 2005; Goecks, Nekrutenko & Taylor, 2010). The remaining sequences were clustered into molecular operational taxonomic units (MOTUs) based on 97% similarity using QIIME 1.8.10 (Caporaso et al., 2010) and filtered to remove infrequent haplotypes (see details in Trevelline et al., 2016). Representative sequences from each MOTU were queried in the Barcode of Life Database (BOLD; Ratnasingham & Hebert, 2007) and scored based on taxonomic resolution and match to a reference sequence (see details in Trevelline et al., 2016). To minimize the likelihood of taxonomic misidentifications from short fragments (157 bp) of the full-length (658 bp) COI barcoding region, MOTUs that exhibited <98% similarity to a reference sequence or could not provide genus- or species-level resolution were classified as “unidentified” and excluded from taxonomic descriptions of diet (discussed in Clare et al., 2011). Because the proportion of sequencing reads does not necessarily reflect the relative quantities of prey consumed (Pompanon et al., 2012), the number of reads assigned to each dietary MOTU were transformed into a presence-absence dataset, which was used to calculate dietary MOTU frequency of occurrence (number of fecal samples in which a MOTU was detected divided by the total number of fecal samples) for both nestling and adult waterthrush.

### Diet analysis

We determined the dietary richness of adult and nestling waterthrush based on the total number of MOTUs (including those that were unidentified; discussed in Clare et al., 2011) detected in fecal samples. We used the frequency of occurrence of dietary MOTUs among nestlings and adults (when possible) associated with the same nest to calculate total dietary niche breadth for each nest using Levins’ Index (reciprocal of Simpson’s Index of diversity; Levins, 1968) in the R package *vegan* (Oksanen et al., 2017; function: diversity, index = “invsimpson”). Levins’ Index of dietary niche breadth was standardized based on the total number of MOTUs in the diets of waterthrush to generate a value ranging from 0 to 1, where 1 represents a diet consisting of all detected MOTUs (Hurlbert, 1978). Taxonomic dietary descriptions were summarized by frequency of occurrence at the order and MOTU level. Identified dietary MOTUs with an aquatic larval stage (hereafter “aquatic prey taxa”) and those without an aquatic larval stage (hereafter “terrestrial prey taxa”) were classified as such using genus-level life history characteristics provided by Merritt & Cummins (2008).

To test the hypothesis that Louisiana Waterthrush shift their diets in response to disturbances in stream quality, changes in dietary MOTU richness and Levins’ Index of niche breadth in response to stream pH and EPT availability were analyzed using linear mixed effect models (LMMs) in the R package *lme4* (Bates et al., 2015; function: lmer). LMMs included random terms to account for the clustering of nests/territories on the same stream and fecal samples associated with the same nest. The dietary niches of waterthrush occupying territories that differed in pH and EPT availability were visualized using non-metric multidimensional scaling (NMDS; Kruskal, 1964) in *vegan* (Oksanen et al., 2017; function: metaMDS, distance = “jaccard”, *k* = 2), which generates a two-dimensional unconstrained ordination plot that illustrates compositional differences between individual diets using minimum convex polygons (function: ordihull) and 95% confidence ellipses around species centroids (function: ordiellipse, kind = “se”, conf = 0.95). To provide additional evidence that waterthrush are vulnerable to changes in stream quality due to dependence on aquatic prey, we applied these same linear and NMDS models to investigate dietary shifts in the nestlings of two syntopic Neotropical migratory species—Acadian Flycatcher (*Empidonax virescens*) and Wood Thrush (*Hylocichla mustelina*)—nesting in the same riparian habitat but are known to primarily consume terrestrial arthropods (data from Trevelline et al., 2018).

We tested whether the presence of all identified dietary MOTUs was predicted by EPT availability (% EPT) using logistic regression. Instead of using separate regressions to individually model the presence of each MOTU, we used a single logistic generalized linear mixed model (GLMM) with separate slopes for each MOTU fitted using random effects (Gelman & Hill, 2007; Harrison et al., 2018). Therefore, in addition random intercepts for streams and nests as in our previous models, we also fitted random intercepts and slopes for each identified MOTU and random intercepts for each diet sample. We then extracted estimated MOTU-specific regression slopes (log-odds ratios; Rita & Komonen, 2008) from the model (Best Linear Unbiased Predictions, BLUPs; Robins, 1989) and their estimated standard errors in the R package *arm* (Gelman & Su, 2016; function: se.coef). We approximated 95% confidence intervals around the BLUPs as ± 1.96*SE.

Estimating MOTU-specific relationships with % EPT via a single GLMM allows us to examine the direction and strength of these relationships with a single-cohesive model, an approach that is becoming increasing popular for multi-species studies (e.g., Jackson et al., 2012; Latta et al., 2017). Fitting unique slopes for each species using random effects generally increases power in a manner analogous to meta-analysis (Qian et al., 2010) by making use of all available data while properly accounting for the correlation among species (MOTUs in our study) originating from the same sample (Chaves, 2010; Davies & Gray, 2015; Harrison et al., 2018). This approach also reduces Type-I errors relative to running separate regressions for each species by attenuating effects sizes closer to zero when relationships are poorly supported by the data, a property known as shrinkage (Halstead et al., 2012). This latter feature of mixed effects models also eliminates the need for correcting for multiple comparison, which would dramatically widen confidence intervals if we ran separate regressions for each MOTU and carried out a Bonferonni-type correction (Gelman, Hill & Yajima, 2012).

## Results

We successfully sequenced COI amplicons from 78 nestling (representing 10 nests) and 14 adult (breeding pairs from seven of the 10 nests) Louisiana Waterthrush fecal samples collected from individuals occupying territories differing in pH (mean of 6.46 ± 0.73 SD, ranging from 4.6 to 7.1) and EPT availability (mean of 0.18 ± 0.06 SD, ranging from 0.07 to 0.27; Data S1). Illumina sequencing generated 7.8 million COI amplicon sequences that reduced to 1.7 million (mean of 18,401 per sample ± 10,082 SD) and 254 MOTUs after quality filtering (Q30), trimming, and removal of infrequent haplotypes. Identification of MOTU representative sequences (Data S2) in the BOLD reference library resulted in ≥98% match to genus or species for 122 MOTUs (48% of total MOTUs; Data S3) representing 94 unique dietary taxa (Table 1).

**Table 1:** Percent frequency of occurrence of identified arthropod MOTUs in the diets of adult and nestling Louisiana Waterthrush. Shading indicates dietary taxa with an aquatic larval stage. Percent frequency of occurrence = number of fecal samples in which a taxon was detected divided by the total number of adult and/or nestling fecal samples.

Class	Order	Family	Genus	Species	% Frequency of occurrence		
					Overall (*n* = 92)	LOWA adults (*n* = 14)	LOWA nestlings (*n* = 78)
Arachnida	Araneae	Lycosidae	Piratula	*Insularis*	5.4	7.1	5.1
		Philodromidae	Philodromus	*Rufus*	5.4	7.1	5.1
	Trombidiformes	Protziidae	Protzia	sp.	6.5	7.1	6.4
Insecta	Blattodea	Cryptocercidae	Cryptocercus	*Punctulatus*	8.7		10.3
	Coleoptera	Curculionidae	Sciaphilus	*Asperatus*	7.6		9.0
	Diptera	Chironomidae	Krenopelopia	sp.	51.1	42.9	52.6
		Culicidae	Anopheles	sp.	20.7	35.7	17.9
		Dolichopodidae	Gymnopternus	*Spectabilis*	8.7	21.4	6.4
		Empididae	Rhamphomyia	sp.	10.9	7.1	11.5
		Limoniidae	Austrolimnophila	*Toxoneura*	5.4	21.4	2.6
			Euphylidorea	*Adustoides*	5.4	14.3	3.8
			Eutonia	*Alleni*	21.7	14.3	23.1
			Limnophila	*Rufibasis*	18.5	35.7	15.4
			Limonia	*Indigena*	22.8	28.6	21.8
			Metalimnobia	*Immatura*	10.9	7.1	11.5
			Rhipidia	*Maculata*	14.1	14.3	14.1
		Pediciidae	Pedicia	sp.	10.9	14.3	10.3
			Tricyphona	*Katahdin*	15.2	21.4	14.1
		Rhagionidae	Symphoromyia	*Fulvipes*	6.5		7.7
		Sciaridae	Schwenckfeldina	*Quadrispinosa*	5.4		6.4
		Stratiomyidae	Allognosta	*Fuscitarsis*	7.6	14.3	6.4
		Syrphidae	Somula	*Decora*	8.7	14.3	7.7
			Temnostoma	*Alternans*	13.0	14.3	12.8
				sp.	5.4		6.4
			Xylota	*Quadrimaculata*	6.5	14.3	5.1
		Tabanidae	Chrysops	sp.	5.4	21.4	2.6
			Hybomitra	*Pechumani*	5.4		6.4
				sp.	28.3	35.7	26.9
			Tabanus	sp.	13.0	21.4	11.5
		Tachinidae	Blepharomyia	*Tibialis*	14.1	14.3	14.1
			Compsilura	*Concinnata*	10.9	14.3	10.3
		Tipulidae	Ctenophora	*Dorsalis*	8.7	7.1	9.0
			Dolichopeza	*Subvenosa*	14.1	14.3	14.1
			Tipula	*Duplex*	5.4		6.4
				*Hermannia*	66.3	92.9	61.5
				*Longiventris*	14.1	21.4	12.8
				*Oropezoides*	16.3	28.6	14.1
				sp.	48.9	57.1	47.4
	Ephemeroptera	Ameletidae	Ameletus	*Lineatus*	34.8	50.0	32.1
				sp.	7.6	7.1	7.7
		Baetidae	Baetis	*Phoebus*	7.6	14.3	6.4
				sp.	7.6	14.3	6.4
		Ephemerellidae	Ephemerella	*Dorothea*	47.8	57.1	46.2
			Eurylophella	*Funeralis*	7.6	7.1	7.7
		Ephemeridae	Ephemera	*Guttulata*	23.9		28.2
		Heptageniidae	Cinygmula	*Subaequalis*	26.1	35.7	24.4
			Epeorus	*Pleuralis*	28.3	42.9	25.6
			Maccaffertium	*Ithaca*	5.4		6.4
				*Pudicum*	21.7	21.4	21.8
		Isonychiidae	Isonychia	sp.	43.5	42.9	43.6
	Hemiptera	Alydidae	Nariscus	*Fumosus*	6.5	28.6	2.6
		Miridae	Neolygus	*Omnivagus*	8.7	14.3	7.7
	Hymenoptera	Tenthredinidae	Craterocercus	*Obtusus*	10.9	14.3	10.3
	Lepidoptera	Erebidae	Hypena	*Baltimoralis*	12.0	21.4	10.3
			Pharga	*Pholausalis*	5.4	28.6	1.3
		Gelechiidae	Chionodes	*Pereyra*	17.4	28.6	15.4
		Geometridae	Ectropis	*Crepuscularia*	16.3	21.4	15.4
			Eupithecia	*Columbiata*	5.4		6.4
			Lomographa	sp.	10.9	21.4	9.0
			Speranza	*Pustularia*	5.4		6.4
		Noctuidae	Anathix	*Ralla*	12.0	21.4	10.3
			Eupsilia	sp.	25.0	21.4	25.6
			Lithophane	sp.	6.5		7.7
			Orthodes	*Cynica*	6.5	14.3	5.1
			Orthosia	*Rubescens*	76.1	85.7	74.4
		Nymphalidae	Calisto	*Aquilum*	7.6		9.0
		Tortricidae	Dichrorampha	*Petiverella*	6.5	7.1	6.4
			Pseudexentera	sp.	28.3	21.4	29.5
				*Spoliana*	9.8	7.1	10.3
	Mecoptera	Bittacidae	Bittacus	*Pilicornis*	8.7	14.3	7.7
	Megaloptera	Corydalidae	Nigronia	*Fasciatus*	41.3	50.0	39.7
				*Serricornis*	27.2	28.6	26.9
	Orthoptera	Rhaphidophoridae	Euhadenoecus	*Puteanus*	23.9	28.6	23.1
	Plecoptera	Capniidae	Arsapnia	*Coyote*	12.0	28.6	9.0
		Chloroperlidae	Alloperla	sp.	12.0	14.3	11.5
				*Usa*	14.1	7.1	15.4
			Haploperla	*Brevis*	12.0	28.6	9.0
			Sweltsa	sp.	13.0	21.4	11.5
		Leuctridae	Leuctra	sp.	46.7	64.3	43.6
		Nemouridae	Amphinemura	sp.	5.4	7.1	5.1
		Perlidae	Acroneuria	*Carolinensis*	60.9	57.1	61.5
		Perlodidae	Clioperla	*Clio*	25.0	35.7	23.1
			Isoperla	sp.	37.0	64.3	32.1
		Pteronarcyidae	Pteronarcys	*Proteus*	32.6	28.6	33.3
	Psocodea	Caeciliusidae	Valenzuela	*Flavidus*	5.4	14.3	3.8
		Peripsocidae	Peripsocus	*Subfasciatus*	6.5	21.4	3.8
	Trichoptera	Goeridae	Goera	*Stylata*	23.9	42.9	20.5
		Limnephilidae	Limnephilus	*Stigma*	18.5	35.7	15.4
			Pycnopsyche	*Gentilis*	13.0	7.1	14.1
				sp.	5.4	7.1	5.1
		Phryganeidae	Ptilostomis	*Ocellifera*	8.7		10.3
		Rhyacophilidae	Rhyacophila	*Minora*	7.6	21.4	5.1
				*Nigrita*	9.8	7.1	10.3
Malacostraca	Decapoda	Cambaridae	Cambarus	sp.	48.9	57.1	47.4

**Figure 1:**
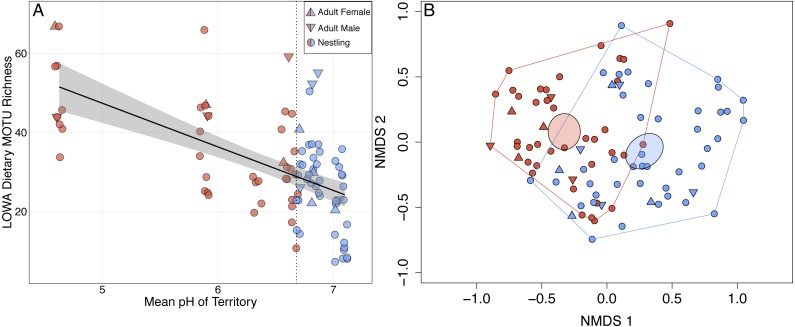
Shifts in adult and nestling Louisiana Waterthrush (LOWA) diet in response to stream acidification. (A) MOTU richness of adult (females, triangles; males, inverted triangles) and nestling (circles) diets increased significantly as mean territory pH declined (${X}_{5,6}^{2}=10.80$; *P* = 0.001). Point shading indicates whether a fecal sample was collected from a territory with a pH ≤ (red) or > (blue) the median value of 6.68 (vertical dotted line). Gray shading represents the 95% confidence interval. (B) Unconstrained NMDS ordination (stress = 0.255) of adult (females, triangles; males, inverted triangles) and nestling (circles) diet composition at the MOTU level. Points represent the taxonomic composition of waterthrush diets and shading indicates that the individual occupied a territory with a pH ≤ (red) or > (blue) the median value of 6.68. Ellipses represent 95% confidence intervals (based on standard error) for group centroids and minimum convex polygons indicate the extent of dietary niche space for each group.

Louisiana Waterthrush dietary richness ranged from 7 to 67 MOTUs (mean of 31.5 per sample ± 13.4 SD; Data S1) and increased significantly as mean territory pH declined (${X}_{\text{5,6}}^{2}=10.80$; *P* = 0.001; Fig. 1A). This trend was observed for both adults (${X}_{\text{4,5}}^{2}=4.97$; *P* = 0.026) and nestlings (${X}_{\text{5,6}}^{2}=11.72$; *P* < 0.001). NMDS analysis revealed that the diets of Louisiana Waterthrush occupying territories with reduced stream pH were distinct from conspecifics in more circumneutral territories (non-overlapping 95% CI ellipses around centroids; Fig. 1B). In contrast, the dietary MOTU richness of Acadian Flycatcher (${X}_{\text{4,5}}^{2}=0.16$; *P* = 0.69; *n* = 44; Figs. S2A and S2B) and Wood Thrush (${X}_{\text{4,5}}^{2}=1.14$; *P* = 0.29; *n* = 51; Figs. S2C and S2D) nestlings occupying the same riparian habitat were unaffected by stream acidification. Like dietary MOTU richness, the total dietary niche breadth (waterthrush nestlings and adults associated with the same nest) increased significantly as mean territory pH declined (${X}_{\text{4,5}}^{2}=4.05$; *P* = 0.044; Fig. S1A).

**Figure 2:**
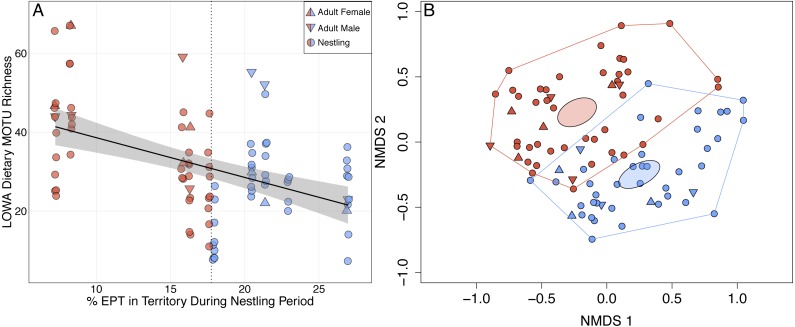
Shifts in adult and nestling Louisiana Waterthrush (LOWA) diet in response to reduced availability of EPT taxa during the period of nestling care. (A) MOTU richness of adult (females, triangles; males, inverted triangles) and nestling (circles) diets increased significantly as percent EPT declined (${X}_{4,5}^{2}=4.97$; *P* = 0.026). Point shading indicates whether a fecal sample was collected from a territory with a percent EPT ≤ (red) or > (blue) the median value of 17.7 (vertical dotted line). Gray shading represents the 95% confidence interval. (B) Unconstrained NMDS ordination (stress = 0.260) of adult (females, triangles; males, inverted triangles) and nestling (circles) diet composition at the MOTU level. Points represent the taxonomic composition of individual diets and shading indicates that the individual occupied a territory with a percent EPT ≤ (red) or > (blue) the median value of 17.7. Ellipses represent 95% confidence intervals (based on standard error) for group centroids and minimum convex polygons indicate the extent of dietary niche space for each group.

Louisiana Waterthrush Dietary MOTU richness increased significantly as percent EPT taxa declined (${X}_{\text{4,5}}^{2}=4.97$; *P* = 0.026; Fig. 2A). This trend was significant for both adults (${X}_{\text{4,5}}^{2}=5.52$; *P* = 0.019) and nestlings (${X}_{\text{5,6}}^{2}=4.64$; *P* = 0.031). NMDS analysis revealed that the diets of waterthrush in riparian habitats with reduced EPT availability were distinct from conspecifics with greater EPT availability (Fig. 2B). In contrast, the dietary MOTU richness of Acadian Flycatcher (${X}_{\text{4,5}}^{2}=0.12$; *P* = 0.73; Figs. S3A and S3B) and Wood Thrush (${X}_{\text{4,5}}^{2}=2.98$; *P* = 0.084; Figs. S3C and S3D) nestlings was unaffected by reduced EPT availability. An increase in total dietary niche breadth of waterthrush nests in response to reduced EPT availability were marginally significant (${X}_{\text{4,5}}^{2}=3.62$; *P* = 0.057; Fig. S1B).

Overall, 16 orders and 50 families of arthropods were detected across nestling and adult waterthrush diets (Table 1). Lepidoptera (100%) and Diptera (97%) were the most frequently detected arthropod orders in both nestling and adult diets (Table 2). Similarly, the pollution-sensitive aquatic orders Ephemeroptera (99%), Plecoptera (91%), and Trichoptera (63%) were among the most frequently detected taxa in both nestling and adult diets (Table 2). Terrestrial dietary MOTU richness (total number of identified taxa without an aquatic life-stage) increased significantly as stream pH (${X}_{\text{5,6}}^{2}=9.24$; *P* = 0.002) and EPT availability (${X}_{\text{5,6}}^{2}=5.83$; *P* = 0.016) declined.

**Table 2:** Percent frequency of occurrence of identified arthropod prey (summarized by order) in the diets of adult and nestling Louisiana Waterthrush.

Class	Order	% Frequency of occurrence		
		Overall (*n* = 92)	LOWA adults (*n* = 14)	LOWA nestlings (*n* = 78)
Insecta	Lepidoptera	100	100	100
Insecta	Ephemeroptera	99	100	99
Insecta	Diptera	97	100	96
Insecta	Plecoptera	91	100	90
Insecta	Megaloptera	64	79	62
Insecta	Trichoptera	63	79	60
Malacostraca	Decapoda	49	57	47
Insecta	Orthoptera	24	29	23
Insecta	Hemiptera	15	43	10
Insecta	Psocodea	12	36	8
Arachnida	Araneae	11	14	10
Insecta	Hymenoptera	11	14	10
Insecta	Blattodea	9	0	10
Insecta	Mecoptera	9	14	8
Insecta	Coleoptera	8	0	9
Arachnida	Trombidiformes	7	7	6

**Figure 3:**
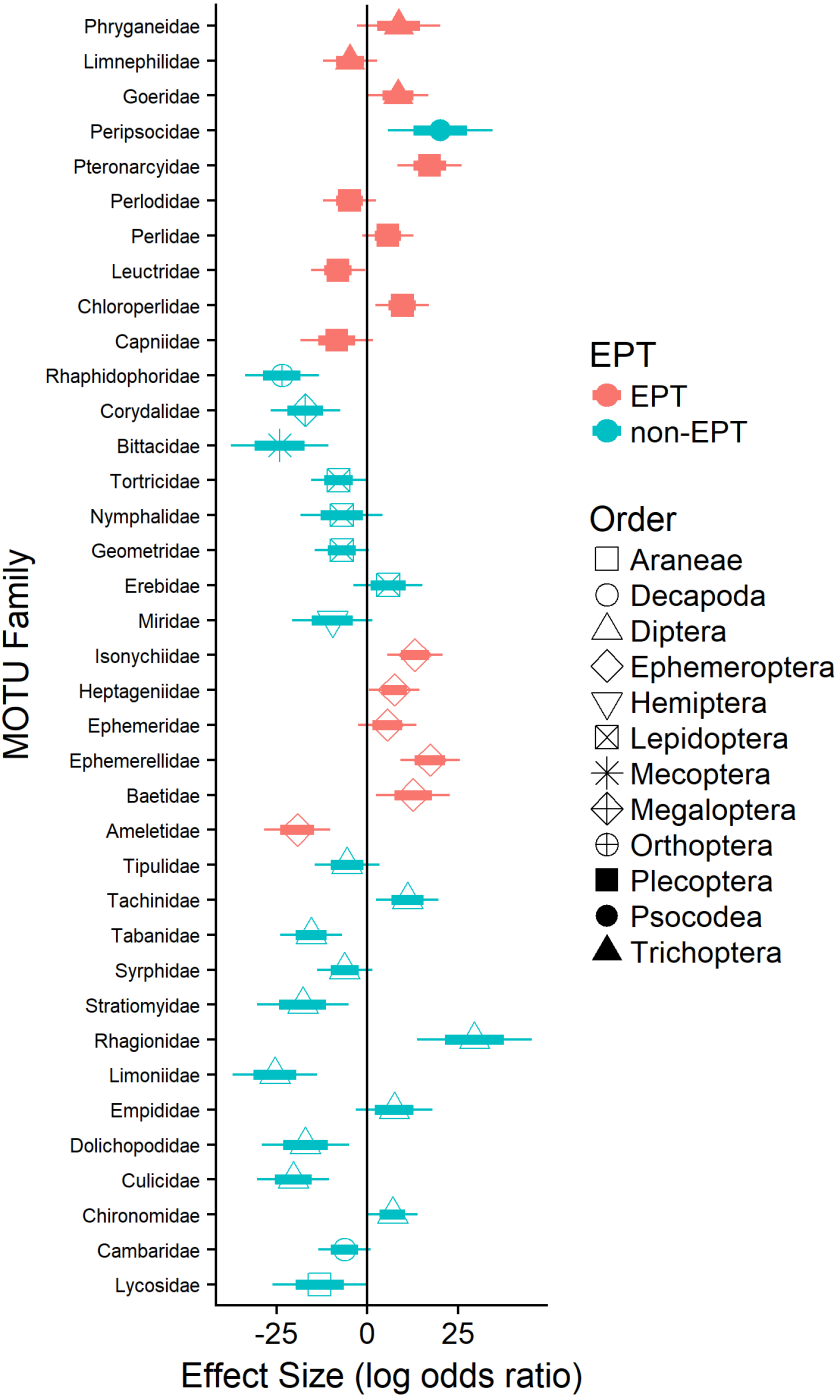
Effect of EPT availability on the occurrence of identified arthropod MOTUs (summarized by family) in the diets of adult and nestling Louisiana Waterthrush nestlings. Only families with significant (*P* ≤ 0.05) increases or decreases in probability are reported. Short bars represent +/ −1 SE; long bars represent +/ −95% confidence intervals.

Logistic regression revealed that the probability of detecting terrestrial arthropods in the orders Araneae (Lycosidae; 11%), Diptera (e.g., Dolichopodidae), Lepidoptera (e.g., Geometridae), Mecoptera (Bittacidae), and Orthoptera (Rhaphidophoridae; 24%) increased significantly as the availability of EPT taxa declined (Fig. 3). Similarly, the probability of detecting pollution-tolerant aquatic arthropods in the orders Diptera (e.g., Culicidae) and Decapoda (Cambaridae) increased significantly as the availability of EPT taxa declined (Fig. 3). In general, the probability of detecting EPT taxa in waterthrush diets decreased as their availability declined (e.g., most Ephemeroptera; Fig. 3); however, the probability of detecting several pollution-sensitive EPT taxa increased significantly (e.g., Ameletidae; Fig. 3) despite their absence from benthic and emergent insect samples collected in acidified territories (Data S4).

## Discussion

In this study, we demonstrated that both adult and nestling Louisiana Waterthrush exhibit dietary shifts in response to stream acidification and reduced EPT availability. These shifts were primarily driven by an expansion of dietary richness (Figs. 1 and 2) and niche breadth (Fig. S1) resulting from the consumption of terrestrial arthropods such as camel crickets (Orthoptera: Rhaphidophoridae), hangingflies (Mecoptera: Bittacidae), moths (Lepidoptera: Geometridae), and wolf spiders (Araneae: Lycosidae; Fig. 3). Therefore, these results provide support for our hypothesis that Louisiana Waterthrush compensate for reduced aquatic prey availability by targeting terrestrial arthropods. In contrast, dietary shifts were not observed for other species of Neotropical migrants (Figs. S2 and S3) nesting alongside waterthrush in the same riparian habitats, but are known to consume primarily terrestrial taxa (Trevelline et al., 2018). These results suggest that the specialized aquatic foraging strategy utilized by Louisiana Waterthrush renders this species vulnerable to disturbances that compromise stream quality and the availability of pollution-sensitive aquatic insects.

While Neotropical migratory birds are known to shift their diets in response to natural fluctuations in prey availability (e.g., Morse, 1978; Rodenhouse & Holmes, 1992; Rosenberg, Ohmart & Anderson, 1982; Rotenberry, 1980) and experimental prey reductions (e.g., Cooper et al., 1990; Rodenhouse & Holmes, 1992; Sample, Cooper & Whitmore, 1993; Whitmore, Cooper & Sample, 1993), our study is the first to demonstrate that this phenomenon can occur as a result of anthropogenic activities that reduce stream pH or otherwise alter aquatic insect community composition. The observed increase in dietary MOTU richness and dietary niche breadth suggests that waterthrush compensate for the loss of preferred EPT taxa (see Trevelline et al., 2016; Trevelline et al., 2018) by altering their provisioning behavior. This explanation is consistent with previous studies demonstrating that Louisiana Waterthrush breeding in habitats with reduced pH and availability of EPT taxa maintain larger territories and expand their foraging areas to include unimpacted peripheral streams (Mulvihill, Newell & Latta, 2008). The expansion of foraging territories in response to habitat degradation has been observed in other species of Neotropical migrants (e.g., Hunter & Witham, 1985; Moulding, 1976) and in stream-dependent dippers (genus *Cinclus*; Feck & Hall, 2004; O’Halloran et al., 1990). For waterthrush, an expansion of foraging areas may provide access to preferred EPT taxa from alternative sources, thereby enabling individuals nesting along acidified streams to tolerate prey limitations within their core territory. This explanation is also supported by our data showing that waterthrush nestlings in acidified territories consumed several acid-sensitive EPT families (e.g., Ameletidae; Fig. 3) that were absent from emergent and benthic insect samples (Data S4), suggesting that adults were provisioning outside of their core territory.

Our results provide evidence that Louisiana Waterthrush are capable of compensating for reduced prey availability by targeting terrestrial arthropods, thereby expanding their dietary niche; however, arthropods of terrestrial origin are generally considered low-quality prey compared to emergent aquatic insects (Twining et al., 2016). Therefore, such dietary shifts have the potential to negatively impact nestling performance or adult reproductive output. For example, experimentally reduced availability of Lepidoptera larvae (preferred prey of most Neotropical migrants during nest provisioning; Holmes, Schultz & Nothnagle, 1979) resulted in a 3–5 day delay in clutch initiation for breeding Red-eyed Vireos (*Vireo olivaceus*), thereby reducing the annual breeding productivity of females (Marshall et al., 2002). Similarly, Rodenhouse & Holmes (1992) demonstrated that Black-throated Blue Warblers (*Setophaga caerulescens*) breeding in plots with reduced Lepidoptera availability attempted fewer nests, thus resulting in fewer fledglings per year. Furthermore, changes in foraging behavior due to reduced prey availability are associated with negative impacts to nestling physiology (Whitmore, Cooper & Sample, 1993) and survival (Nagy & Smith, 1997). Because the expansion of territories has been shown to increase foraging effort and reduce parental care (O’Halloran et al., 1990), stream-dependent songbirds may be at greater risk for predation (Martin, Scott & Menge, 2000) and brood parasitization (Arcese & Smith, 1988), thus reducing nestling survival in acidified habitats and possibly contributing to current population declines (Martin, 1987).

This study was based on the diets of Louisiana Waterthrush along three streams over the course of a single breeding season. Because diets can vary drastically between locations (e.g., Rotenberry, 1980) and years (see differences in Plecoptera consumption among waterthrush nestlings in Trevelline et al., 2016; Trevelline et al., 2018), the taxonomic composition of diets presented here should not be considered a fully representative description. Furthermore, the use of a single arthropod-specific PCR primer set prevents the detection of vertebrate taxa thought to be provisioned more frequently by waterthrush nesting along acidified streams (e.g., small fish and salamanders; Mattsson et al., 2009; Mulvihill, Newell & Latta, 2008). Despite the exclusion of vertebrate prey that would likely increase the magnitude of the dietary shifts, our approach successfully detected significant differences in waterthrush diets as stream pH and EPT availability declined. It is important to note, however, that DNA metabarcoding cannot differentiate between arthropod life-stages (adult and larval insects have identical COI barcode sequences). Therefore, it is impossible to determine (from our data) if waterthrush occupying acidified habitats may further compensate by targeting emergent aquatic insects rather than aquatic larvae, which most likely differ in nutritional content (e.g., Arrese & Soulages, 2010) and required handling effort (e.g., Sherry & McDade, 1982). Nevertheless, these limitations were consistent across all fecal samples, and therefore should not invalidate our major conclusion that waterthrush alter their diets in response to stream acidification.

## Conclusions

In this study, we provided evidence that stream acidification alters the dietary niche of a Neotropical migratory songbird via disruption of aquatic prey availability. This phenomenon appears to be mediated through the reduced availability of pollution-sensitive EPT taxa, which are vulnerable to a wide-range of anthropogenic activities that affect the chemical or geomorphic profile of aquatic habitats (e.g., Roy et al., 2003; Wood, Frantz & Becker, 2016). Given the increasing frequency and intensity of anthropogenic disturbances in riparian ecosystems (Drohan et al., 2012; Dudgeon et al., 2006) and the known impact of food limitations on the breeding productivity of Neotropical migrants (reviewed in Martin, Scott & Menge, 2000), these activities may negatively impact the conservation of Louisiana Waterthrush or other Neotropical migrants known to opportunistically utilize aquatic insects while breeding in riparian habitats (Trevelline et al., 2018).

##  Supplemental Information

10.7717/peerj.5141/supp-1Figure S1Shifts in adult and nestling Louisiana Waterthrush (LOWA) dietary niche breadth in response to stream acidification and reduced EPT availability during the period of nestling care(A) Total dietary niche breadth (all adults and nestlings associated with a nest) increased significantly (${X}_{4,5}^{2}=4.05$; *P* = 0.04) as mean territory pH declined (vertical dotted line = median territory pH of 6.68). (B) Total dietary niche breadth (all adults and nestlings associated with a nest) exhibited a marginally significant increase (${X}_{4,5}^{2}=3.62$; *P* = 0.057) in response to reduced percent EPT (vertical dotted line = median territory percent EPT of 17.7). Gray shading represents the 95% confidence interval.

10.7717/peerj.5141/supp-2Figure S2The diets of Acadian Flycatcher (ACFL) and Wood Thrush (WOTH) nestlings in riparian habitats are unaffected by stream acidification(A) Dietary MOTU richness of Acadian Flycatcher nestlings did not differ significantly as mean territory pH declined (${X}_{4,5}^{2}=0.16$; * P* = 0.69). Point shading indicates whether a fecal sample was collected from a territory with a pH ≤ (red) or > (blue) the median value of 6.34 (vertical dotted line). Gray shading represents the 95% confidence interval. (B) Unconstrained NMDS ordination (stress = 0.247) of Acadian Flycatcher nestling diet composition at the MOTU level. Points represent the taxonomic composition of individual diets and shading indicates that the individual occupied a territory with a pH ≤ (red) or > (blue) the median value of 6.34. Ellipses represent 95% confidence intervals (based on standard error) for group centroids and minimum convex polygons indicate the extent of dietary niche space for each group. (C) Dietary MOTU richness of Wood Thrush nestlings did not differ significantly as mean territory pH declined (${X}_{4,5}^{2}=1.14$; * P* = 0.29). Point shading indicates whether a fecal sample was collected from a territory with a pH ≤ (red) or > (blue) the median value of 6.24 (vertical dotted line). (D) Unconstrained NMDS ordination (stress = 0.258) of Wood Thrush nestling diet composition at the MOTU level. Points represent the taxonomic composition of individual diets and shading indicates that the individual occupied a territory with a pH ≤ (red) or > (blue) the median value of 6.24.

10.7717/peerj.5141/supp-3Figure S3The diets of Acadian Flycatcher (ACFL) and Wood Thrush (WOTH) nestlings in riparian habitats are unaffected by reduced EPT availability during the period of nestling care(A) Dietary MOTU richness of Acadian Flycatcher nestlings did not differ significantly as percent EPT declined (${X}_{4,5}^{2}=0.12$; *P* = 0.73). Point shading indicates whether a fecal sample was collected from a territory with a percent EPT ≤ (red) or > (blue) the median value of 2.05. (vertical dotted line). Gray shading represents the 95% confidence interval. (B) Unconstrained NMDS ordination (stress = 0.247) of Acadian Flycatcher nestling diet composition at the MOTU level. Points represent the taxonomic composition of individual diets and shading indicates that the individual occupied a territory with a percent EPT ≤ (red) or > (blue) the median value of 2.05. Ellipses represent 95% confidence intervals (based on standard error) for group centroids and minimum convex polygons indicate the extent of dietary niche space for each group. (C) Dietary MOTU richness of Wood Thrush nestlings did not differ significantly as percent EPT declined (${X}_{4,5}^{2}=2.98$; *P* = 0.084). Point shading indicates whether a fecal sample was collected from a territory with a percent EPT ≤ (red) or > (blue) the median value of 1.64 (vertical dotted line). (D) Unconstrained NMDS ordination (stress = 0.260) of Wood Thrush nestling diet composition at the MOTU level. Points represent the taxonomic composition of individual diets and shading indicates that the individual occupied a territory with a percent EPT ≤ (red) or > (blue) the median value of 1.64.

10.7717/peerj.5141/supp-4 Data S1 Sample metadata with presence/absence of denovo OTUs

10.7717/peerj.5141/supp-5 Data S2 MOTU representative sequences for BOLD identification

10.7717/peerj.5141/supp-6 Data S3 BOLD identifications for MOTU representative sequences

10.7717/peerj.5141/supp-7 Data S4 Raw insect availability data from benthic and sticky trap samples

10.7717/peerj.5141/supp-8 Supplemental Information 1 R code for linear mixed effect models

10.7717/peerj.5141/supp-9 Supplemental Information 2 Input diet MOTU richness data for linear mixed effect models (RCodeForLMMs.R) for adult and nestling Louisiana Waterthrush

10.7717/peerj.5141/supp-10 Supplemental Information 3 Input diet MOTU richness data for linear mixed effect models (RCodeForLMMs.R) for adult Louisiana Waterthrush

10.7717/peerj.5141/supp-11 Supplemental Information 4 Input diet MOTU richness data for linear mixed effect models (RCodeForLMMs.R) for nestling Louisiana Waterthrush

10.7717/peerj.5141/supp-12 Supplemental Information 5 Input diet MOTU richness data for linear mixed effect models (RCodeForLMMs.R) summarized by Louisiana Waterthrush nest

10.7717/peerj.5141/supp-13 Supplemental Information 6 Input diet MOTU richness data for linear mixed effect models (RCodeForLMMs.R) for nestling Acadian Flycatcher (data from Trevelline et al., 2018)

10.7717/peerj.5141/supp-14 Supplemental Information 7 Input diet MOTU richness data for linear mixed effect models (RCodeForLMMs.R) for nestling Wood Thrush (data from Trevelline et al., 2018)

10.7717/peerj.5141/supp-15 Supplemental Information 8 Diet MOTUs for logistic regression models Each row represents the identified MOTUS for a given waterthrush fecal sample. These are the raw data are used for the logistic regression models, and the input file for R code in Trevelline_PeerJ_2018_tidy.Rmd

10.7717/peerj.5141/supp-16 Supplemental Information 9 R code for tidying of raw MOTU data for logistic regression models R code for tidying of raw MOTU data. Output file is ”data_mult_spp_glmm.csv” used for logistic regression models.

10.7717/peerj.5141/supp-17 Supplemental Information 10 Output file from R code contained in Trevelline_PeerJ_2018_tidy.Rmd for logistic regression models Output file from R code contained in Trevelline_PeerJ_2018_tidy.Rmd for logistic regression models in RMD file Trevelline_PeerJ_2018_glmm.Rmd.

10.7717/peerj.5141/supp-18 Supplemental Information 11 R code for logistic regression models for diet MOTUs using EPT availability as predictor This code was used to produce Fig. 3.

## Additional Information and Declarations

### Field Study Permissions

The following information was supplied relating to field study approvals (i.e., approving body and any reference numbers): Permissions to complete the work were provided by the Pennsylvania Game Commission, the Pennsylvania Department of Conservation and Natural Resources, and the USGS Bird Banding Lab. Access to study sites and logistical support were provided by the PA Bureau of Forestry, PA Bureau of State Parks, Powdermill Nature Reserve, and private landowners.

### DNA Deposition

The following information was supplied regarding the deposition of DNA sequences: Raw Illumina sequencing data is available from GenBank accession number SRP126286: SRR6780942 –SRR6781033.

### Data Availability

The following information was supplied regarding data availability: The data and R code are provided as Supplemental Files.
