# Supplementary figures and images for: Stream acidification and reduced aquatic prey availability are associated with dietary shifts in an obligate riparian Neotropical migratory songbird
Source: PeerJ. 2018 Jul 6;6:e5141. doi: 10.7717/peerj.5141 (PMC6037135; doi:10.7717/peerj.5141)

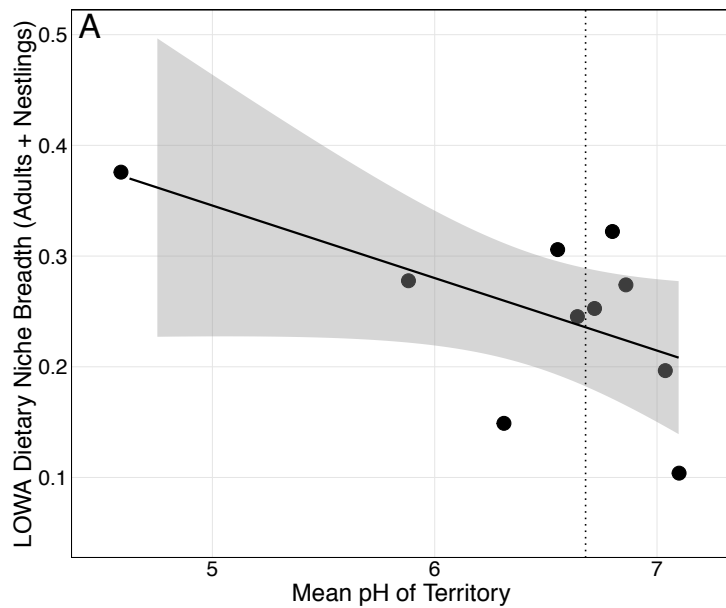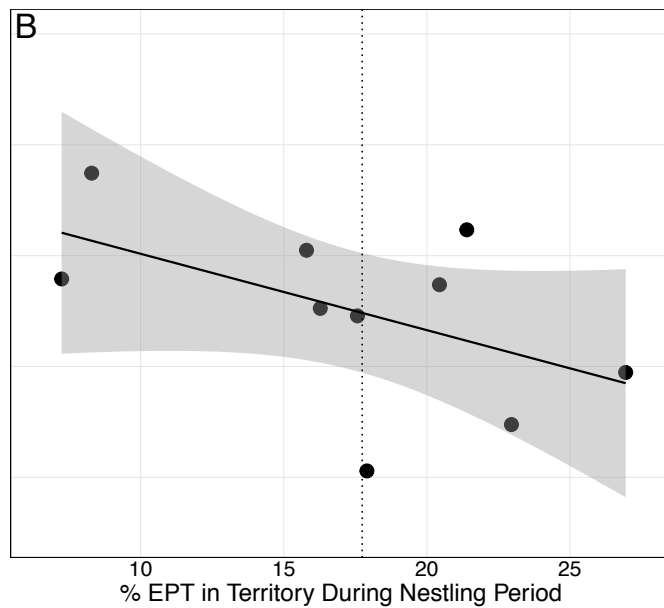

Supplement: Figure S1 — (A) Total dietary niche breadth (all adults and nestlings associated with a nest) increased significantly (${X}_{4,5}^{2}=4.05$X4,52=4.05; P = 0.04) as mean territory pH declined (vertical dotted line = median territory pH of 6.68). (B) Total dietary niche breadth (all adults and nestlings associated with a nest) exhibited a marginally significant increase (${X}_{4,5}^{2}=3.62$X4,52=3.62; P = 0.057) in response to reduced percent EPT (vertical dotted line = median territory percent EPT of 17.7). Gray shading represents the 95% confidence interval. [file peerj-06-5141-s001.pdf]

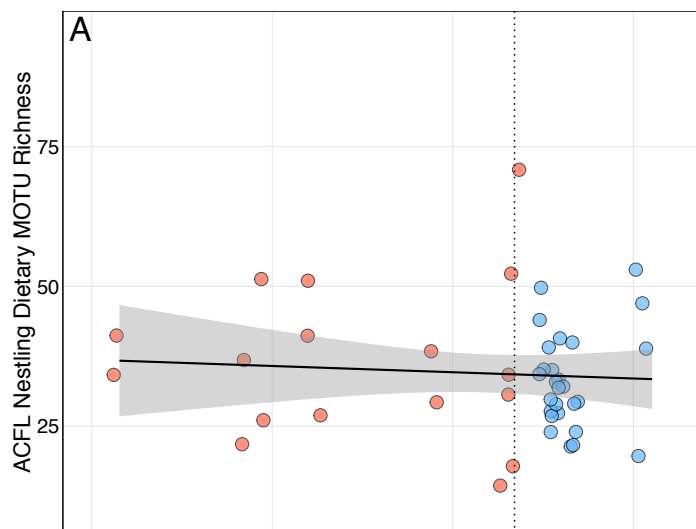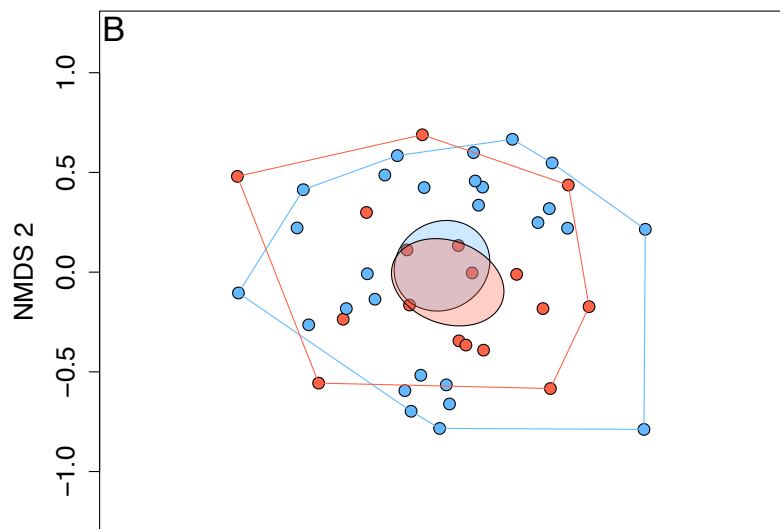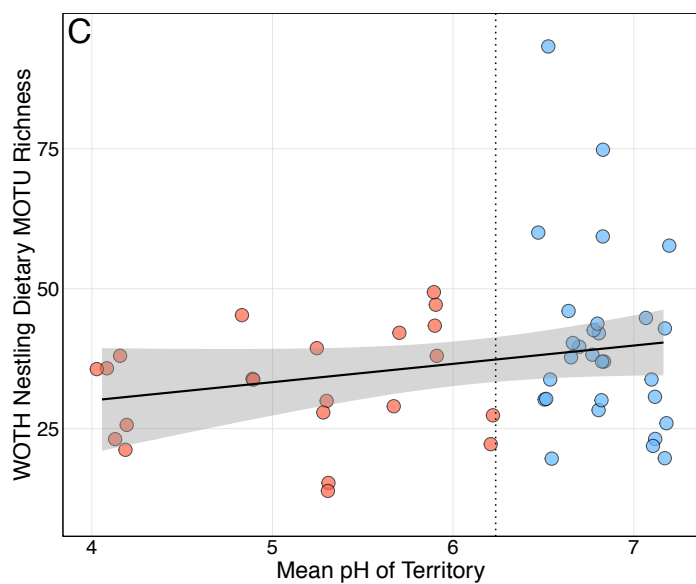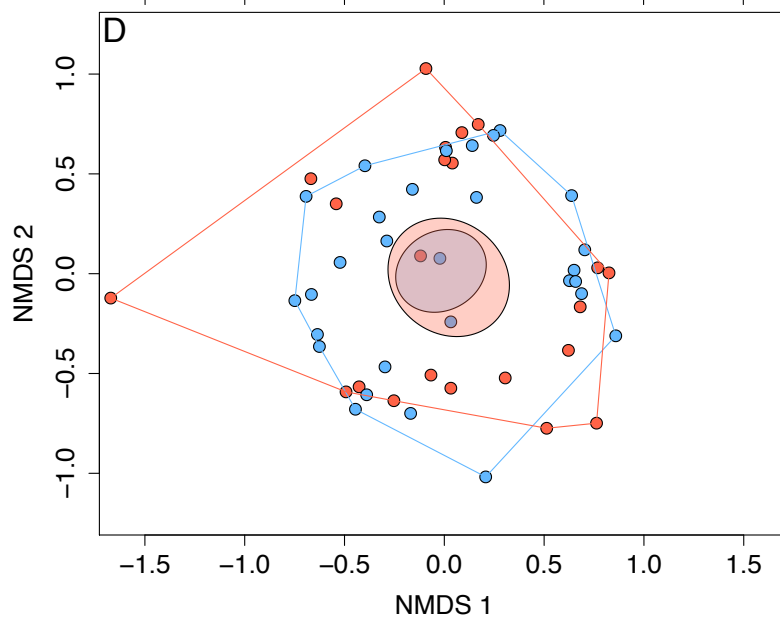

Supplement: Figure S2 — (A) Dietary MOTU richness of Acadian Flycatcher nestlings did not differ significantly as mean territory pH declined (${X}_{4,5}^{2}=0.16$X4,52=0.16; P = 0.69). Point shading indicates whether a fecal sample was collected from a territory with a pH ≤ (red) or > (blue) the median value of 6.34 (vertical dotted line). Gray shading represents the 95% confidence interval. (B) Unconstrained NMDS ordination (stress = 0.247) of Acadian Flycatcher nestling diet composition at the MOTU level. Points represent the taxonomic composition of individual diets and shading indicates that the individual occupied a territory with a pH ≤ (red) or > (blue) the median value of 6.34. Ellipses represent 95% confidence intervals (based on standard error) for group centroids and minimum convex polygons indicate the extent of dietary niche space for each group. (C) Dietary MOTU richness of Wood Thrush nestlings did not differ significantly as mean territory pH declined (${X}_{4,5}^{2}=1.14$X4,52=1.14; P = 0.29). Point shading indicates whether a fecal sample was collected from a territory with a pH ≤ (red) or > (blue) the median value of 6.24 (vertical dotted line). (D) Unconstrained NMDS ordination (stress = 0.258) of Wood Thrush nestling diet composition at the MOTU level. Points represent the taxonomic composition of individual diets and shading indicates that the individual occupied a territory with a pH ≤ (red) or > (blue) the median value of 6.24. [file peerj-06-5141-s002.pdf]

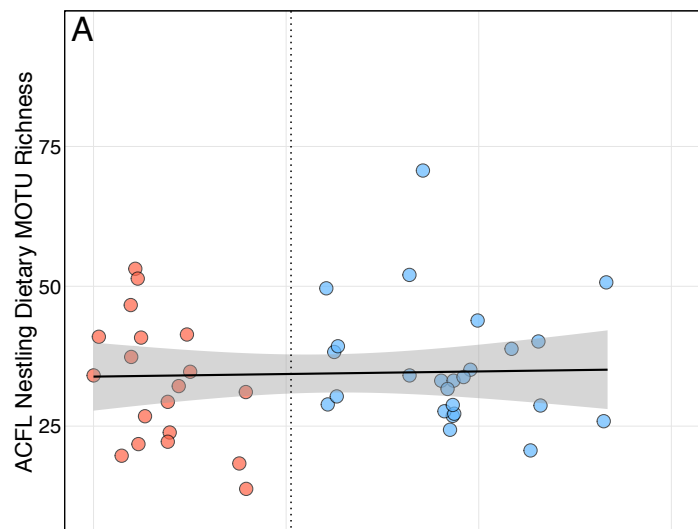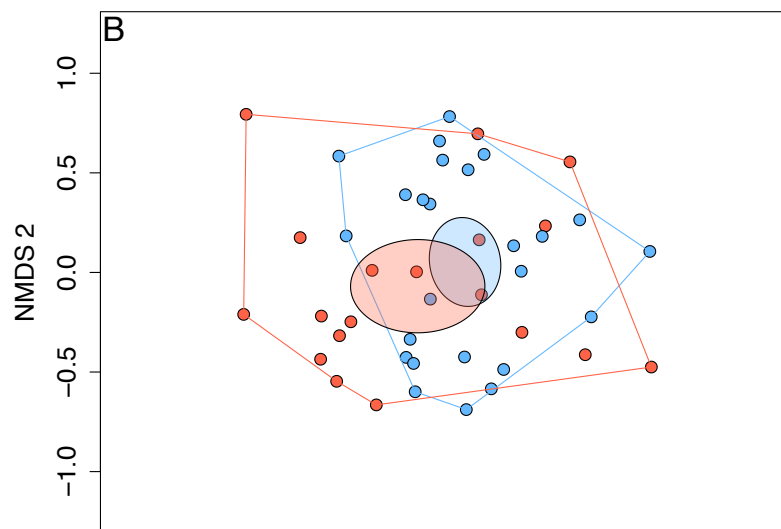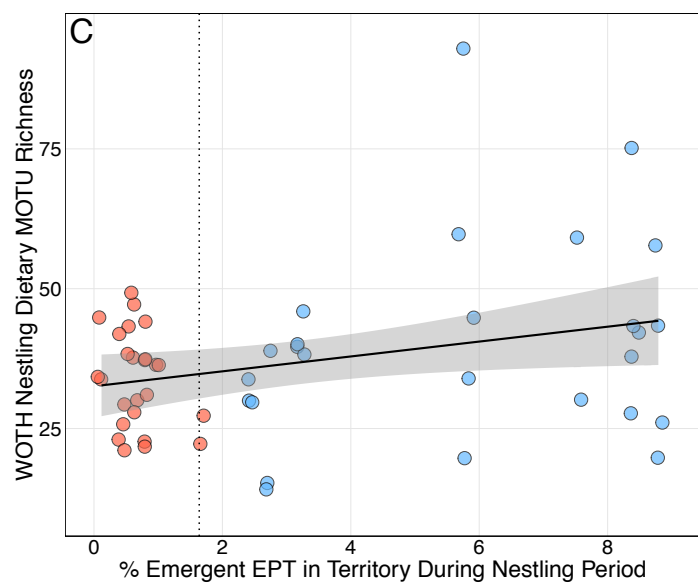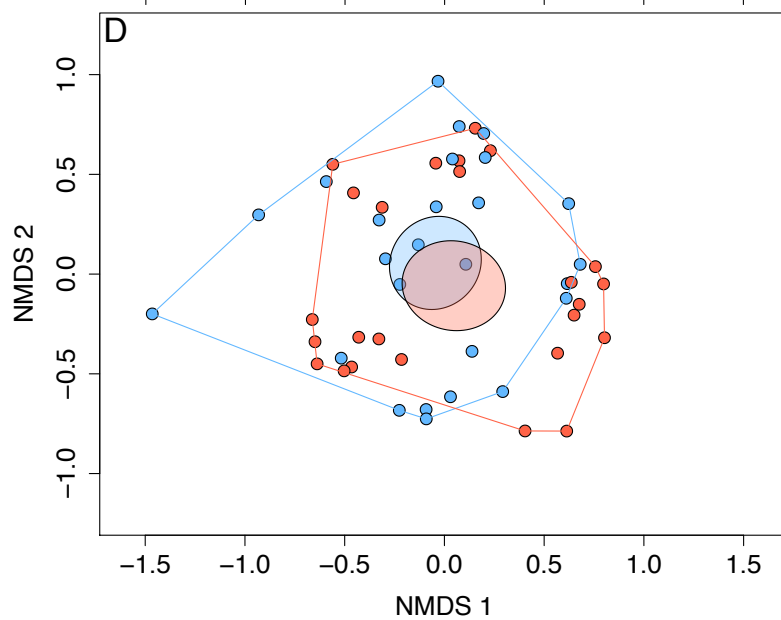

Supplement: Figure S3 — (A) Dietary MOTU richness of Acadian Flycatcher nestlings did not differ significantly as percent EPT declined (${X}_{4,5}^{2}=0.12$X4,52=0.12; P = 0.73). Point shading indicates whether a fecal sample was collected from a territory with a percent EPT ≤ (red) or > (blue) the median value of 2.05. (vertical dotted line). Gray shading represents the 95% confidence interval. (B) Unconstrained NMDS ordination (stress = 0.247) of Acadian Flycatcher nestling diet composition at the MOTU level. Points represent the taxonomic composition of individual diets and shading indicates that the individual occupied a territory with a percent EPT ≤ (red) or > (blue) the median value of 2.05. Ellipses represent 95% confidence intervals (based on standard error) for group centroids and minimum convex polygons indicate the extent of dietary niche space for each group. (C) Dietary MOTU richness of Wood Thrush nestlings did not differ significantly as percent EPT declined (${X}_{4,5}^{2}=2.98$X4,52=2.98; P = 0.084). Point shading indicates whether a fecal sample was collected from a territory with a percent EPT ≤ (red) or > (blue) the median value of 1.64 (vertical dotted line). (D) Unconstrained NMDS ordination (stress = 0.260) of Wood Thrush nestling diet composition at the MOTU level. Points represent the taxonomic composition of individual diets and shading indicates that the individual occupied a territory with a percent EPT ≤ (red) or > (blue) the median value of 1.64. [file peerj-06-5141-s003.pdf]
